# Transport of CCL2 across an induced pluripotent stem cell-derived *in vitro* model of the human blood-brain barrier is heparan sulfate-dependent

**DOI:** 10.1371/journal.pone.0338780

**Published:** 2025-12-11

**Authors:** Lindsey M. Williams, Takashi Fujimoto, Mamatha Damodarasamy, Riley R. Weaver, C. Dirk Keene, William A. Banks, May J. Reed, Michelle A. Erickson

**Affiliations:** 1 Geriatric Research Education and Clinical Center (GRECC), VA Puget Sound Healthcare System, Seattle, Washington, United States of America; 2 Department of Pharmaceutics, University of Washington School of Pharmacy, Seattle, Washington, United States of America; 3 Department of Neurosurgery, Nagasaki University Graduate School of Biomedical Sciences, Nagasaki, Japan; 4 Department of Medicine, Division of Gerontology and Geriatric Medicine, University of Washington School of Medicine, Seattle, Washington, United States of America; 5 Department of Laboratory Medicine and Pathology, Division of Neuropathology, University of Washington, Seattle, Washington, United States of America; Eötvös Loránd Research Network Biological Research Centre, HUNGARY

## Abstract

Transport of immune-active substances across the blood-brain barrier (BBB) is an important mechanism of neuroimmune regulation. CCL2 is an exemplary chemokine that regulates neuroinflammation and can cross the intact BBB from blood to brain in mice. This study aimed to characterize the blood-to-brain transport mechanisms of human CCL2 using human induced pluripotent stem cell (iPSC)-derived brain endothelial-like cells (iBECs), an *in vitro* BBB model. Since heparan sulfate (HS) is an important component of the *in vivo* BBB that regulates CCL2 transport in mice, we first assessed HS deposition in iBECs and found that HS levels increased with extended culture time. We therefore evaluated transport of radiolabeled ^125^I-CCL2 and ^131^I-BSA after nine days in subculture, after HS had sufficient time to accumulate. To determine the predominant transport mechanism *in vitro*, we evaluated whether transport of ^125^I-CCL2 and ^131^I-BSA was altered in the presence of an inhibitor of the chemokine receptor CCR2, and following treatment with heparin, heparinase enzymes, or the heparan sulfate synthesis inhibitor GalNaz which all test HS-dependent mechanisms of transport. We also evaluated the expression of CCR2 and membrane-bound HS proteoglycans (HSPGs) in iBECs and in isolated human brain microvessels. We found that iBECs have a functional blood-to-brain transport system for CCL2. Similar to findings in mice, heparin inhibited CCL2 transport whereas the CCR2 inhibition did not. Both heparinase treatment and treatment with GalNaz inhibited CCL2 transport across the BBB, further supporting the involvement of HS in CCL2 transport. The iBECs expressed CCR2 at levels comparable to human brain microvessels, and had detectable expression of the syndecans 1–4 and glypicans 1–6, whereas human brain microvessels expressed syndecans 2–4 and glypicans 2–5 in all subjects tested. Our findings highlight that iBECs are a useful tool for studying the involvement of heparan sulfate/glycocalyx components in the transport of substances across the BBB.

## Introduction

The vascular blood-brain barrier (BBB) is an important interface for neuroimmune communication [1,2]. Brain endothelial cells (BECs) that primarily comprise the BBB regulate many aspects of bidirectional communication between the brain and immune system; among these is their ability to transport immune active substances such as cytokines and chemokines [1]. Cytokines and chemokines are among the key regulatory proteins of the immune system, and their BBB transport, characterized mostly in mice [3–5], regulates critical CNS functions [6]. For example, BBB transport of IL-1α at the posterior division of the septum regulates memory processing in mice [7] and is implicated in the impairment of memory functions during transient sickness behaviors that arise as a result of immune activation [6]. Blood-to-brain transport of chemokines, a subset of structurally related chemotactic cytokines, has also been demonstrated in rodents using *in vitro* and *in vivo* models [8,9]. Chemokines are best known for their role in leukocyte trafficking [10], but they have also been implicated in aspects of CNS dysfunction that may be leukocyte-independent. For example, CCL11 inhibits neurogenesis and contributes to cognitive impairment with aging [11]. Therefore, the transport of chemokines from blood-to-brain could have important effects on CNS functions both at baseline and during systemic inflammation.

Chemokines can interact with endothelial cells via binding to chemokine receptors, and via interactions with heparan sulfate (HS), an important component of the brain endothelial glycocalyx [12–14]. Chemokine receptors such as CCR2 and the duffy antigen receptor for chemokines (DARC) were shown to be particularly important transport mediators of CCL2 from the brain-to-blood across the BBB [15,16]. Current evidence supports that binding to HS, a glycosaminoglycan that is abundant in the glycocalyx of endothelial cells [14,17], contributes to the blood-to-brain transport of both CCL2 and CCL5 across the intact mouse BBB [9]. Further, HS and other extracellular matrix components do not mediate binding of CCL2 to the abluminal surface of the BBB [18], suggesting that brain-to-blood and blood-to-brain transport of CCL2 and perhaps other chemokines as well are regulated by distinct mechanisms. We posit here that HS is an underexplored component of the brain endothelium that can mediate transport of substances, particularly those known to bind HS, across the BBB. However, existing models and methods for studying HS-mediated BBB transport are currently limited/not widely available, and manipulation of the glycocalyx *in vivo* is both costly and challenging. In the present study, our goal was to determine whether HS-dependent chemokine transport could be modeled and characterized *in vitro* using a well-established iPSC-derived brain endothelial-like cell (iBEC) model of the human BBB.

Although there is evidence that structural recapitulation of the endothelial glycocalyx, particularly its thickness, is better recapitulated in 3D culture with flow [19], we reasoned that a 2D iBEC BBB model could be used to model functional aspects of HS-dependent chemokine transport since the HS that mediates transport is likely attached to a cell surface HS proteoglycan and thus in close proximity to the cell membrane. The 2D model is also better suited for mid-throughput BBB leakage/transport assays using radiochemical tracers, which offer highly quantitative and sensitive detection vs. similarly sized fluorophores, whose leakage and transport across intact iBECs is often below the limit of detection [20]. We first established that HS is present and accumulates on the surface of cultured iBECs. We then showed that blood-to-brain transport of human CCL2 occurs in iBECs, and confirmed its mechanism of transport as being HS-dependent using three methods of HS inhibition, whereas a CCR2 inhibitor at a pharmacologically active concentration had no effect. Finally, we showed that iBECs express both CCR2 and HSPGs, although HSPG expression patterns were slightly different when compared with human brain microvessels. Our results support that CCL2 can cross the intact BBB in the blood-to-brain direction via an HS-dependent mechanism, and highlight that iBECs can be used as a model to study HS-dependent BBB transport.

## Materials and methods

### Ethics statement

This specific study uses only preexisting de-identified human samples and cells and does not meet criteria to be categorized as human subjects research according to United States Federal Policy.

### Derivation of brain endothelial-like cells (iBECs) from hiPSCs and TEER measurements

iBECs were derived using the method of Neal et al [21], with modifications. Human iPSCs (hiPSCs) from the GM25256 line (Coriell Institute) were maintained on plates coated with Matrigel (Corning, cat no. 356230) in E8 Flex medium (Thermo Fisher Scientific, cat no. A28585-01). The day before differentiation was initiated, hiPSCs were dissociated into single cells with Accutase (Thermo Fisher Scientific, cat no. A1110501) and plated onto Matrigel-coated plates at a density of 15 x 10^3^ cells/well in E8 Flex medium supplemented with 10 µM Rho-associated protein kinase (ROCK) inhibitor Y-27632 (R&D Systems, cat no. 1254). To initiate differentiation, the medium was changed to E6 (Thermo Fisher Scientific, cat no. A1516401) and E6 medium changes continued daily for 3 more days. Then, the medium was changed to human endothelial serum-free medium (HESFM, Thermo Fisher Scientific, cat no. 11111044) supplemented with 20 ng/mL basic fibroblast growth factor (bFGF; Peprotech, cat no. 100-18B), 10 µM retinoic acid (RA; Sigma, cat no. R2625), and 1% B27 supplement (Thermo Fisher Scientific, cat no. 17504044). After two days, iBECs were dissociated with Accutase and subcultured onto 24-well transwell permeable inserts (Corning, cat no. 3470) or tissue culture plates (Corning, cat no. 3513, 3548) coated with 1 mg/mL Collagen IV (Sigma, cat no. C5533) and 5 mM Fibronectin (Sigma, cat no. F1141) in HESFM + 20 ng/mL bFGF, 10 µM RA, and 1% B27 (day 0). 24 h later, the medium was changed to HESFM + 1% B27 without bFGF or RA, and resistance (Ω) values for monolayers of iBECs seeded on transwells were obtained for day 1 using an EVOM2 Voltohmmeter (World Precision Instruments, Sarasota Florida) coupled to an ENDOHM cup chamber. Transendothelial electrical resistance (TEER) was calculated by subtracting the resistance (Ω) value of a blank transwell and multiplying by the transwell surface area (0.33 cm^2^). Experiments were only conducted on iBECs with TEER > 1000 Ω*cm^2^. Above this threshold of TEER, the predominant mode of iBEC leakage is via small numbers of pinocytic vesicles, which have an approximate size of 30−100nm [22]. We have shown previously that iBECs cultured using this method express tight junction proteins claudin-5, ZO-1, and occludin as well as endothelial markers PECAM-1 and VE-Cadherin, which persist and become more localized to junctional sites over time in culture [23].

### Immunocytochemistry and immunofluorescence analyses

iBECs plated on 48-well plates were washed once with PBS (Thermo Fisher Scientific, cat no. 70011044) and fixed in a 1:1 methanol/acetone mixture for 15 min at 4°C. Wells were washed with PBS 3x for 5 min each, then blocked with 5% normal donkey serum (Jackson ImmumoResearch, cat no. 017-000-121) + 0.1% TX-100 (Sigma, cat no. X100) in PBS for 1 h at RT. Wells were washed 3x for 5 min each, then iBECs were incubated with 1:100 dilutions of Heparan Sulfate clone F58-10E4 (HS(10E4)) primary antibody (amsbio, cat no. 370255) in phosphate-buffered normal antibody diluent (NAD) (Scytek, cat no. ABB500) overnight at 4°C. Wells were washed 3x for 5 min each, then incubated with 1:200 dilution of secondary antibody (Thermo Fisher, cat no. A-21426) and 1:5000 dilution of DAPI (Thermo Fisher Scientific, cat no. 62248) in NAD for 1 h at RT. Wells were washed 3x for 5 min each, then imaged using a Zeiss Axiovert 7. For each differentiation, 3 wells were designated per group and 3 images were taken per well. Mean fluorescence intensity (MFI) values were quantified using the Zen image analysis software and were corrected for differences in cell density via normalization to DAPI area.

### Radioactive iodination of CCL2 and albumin

The chloramine-T method was used to radioactively label recombinant carrier-free human CCL2 (R & D Systems, 279-MC-050/CF) with ^125^I and bovine serum albumin (BSA, Sigma cat no. 17030-100G) with ^131^I, as described previously [24]. 10 µg of chloramine-T (Sigma, cat no. C-9887) was incubated with 5 µg of CCL2 and 0.5 mCi of ^125^I (carrier-free, Perkin Elmer) for CCL2 labeling or 100 µg of BSA and 2 mCi of ^131^I (carrier-free, GE) for BSA labeling. The reactions occurred for 1 minute in 0.25 M phosphate buffer then were terminated by an addition of 100 µg of sodium metabisulfite (Sigma, cat no. S9000). The iodinated proteins were separated from free iodine by purification on a column of G-10 Sephadex.

### CCL2 transport assays

Transwells were distributed such that the mean TEER values were approximately equal among groups. The luminal and abluminal mediums were refreshed with fresh heparin-free DMEM/F-12 (Thermo Fisher Scientific, cat no. 11330032)+ 1% B27, and cells were equilibrated at 37°C for 20 min. To initiate the assay, the 100 µL luminal chamber volume was switched to an input medium consisting of DMEM/F-12 + 1% B27 containing 3 million counts per minute (CPM) of ^125^I-CCL2 and 3 million CPM ^131^I-albumin (^131^I-Alb) per Transwell. ^131^I-Alb was included to quantify non-specific leakage pathways, which are predominantly vesicular in iBECs with high TEER. As albumin and CCL2 diameters (about 4.5−22 nm, depending on orientation) are smaller than the size of a pinocytic vesicle (30−100nm) [22], their passage across iBECs due to non-specific vesicular leakage pathways is expected to be similar. To evaluate the effects of heparin on CCL2 transport, 20 U/mL of heparin (Sigma, cat no. H3149) was included in the input medium. To evaluate the effects of CCR2 inhibition on CCL2 transport, 10 µM of RS504393 (Sigma, cat no. SML07011) was included in the input medium. After incubation times of 15, 30, 45, 60, and 90 min. at 37°C, 500 µL volumes of medium from the abluminal chamber were collected and replaced with fresh, pre-warmed DMEM/F-12 + 1% B27 medium. Collections were mixed with 200 µL of 1% BSA in lactated Ringer’s solution and 15% trichloroacetic acid to precipitate the intact proteins in solution, then centrifuged at 4255g for 15 min to pellet the proteins. The supernatant containing free iodine and degraded proteins was discarded. The radioactivity in the pellet was counted in a Wizard 2 gamma counter (Perkin Elmer) to measure ^125^I-CCL2 and ^131^I-Alb levels. Permeability-surface area coefficient (Pe) calculations were performed according to the method of Dehouck et al. [25]. Clearance was expressed as nL of radioactive tracer transported from the luminal chamber to the abluminal chamber, and was calculated from the initial level of acid-precipitable radioactivity added to the luminal chamber and the final level of radioactivity in the abluminal chamber:


$$\text{Clearance}\;(\mu \text{L})=[\text{C}{]}_{\text{c}}\times {\text{V}}_{\text{c}}/[\text{C}{]}_{\text{L}}$$

Where [C]_L_ is the initial concentration of radioactivity in the luminal chamber (in units of CPM/µL), [C]_C_ is the concentration of radioactivity in the abluminal chamber (in units of CPM/µL) and Vc is the volume of the abluminal chamber in µL. The volume cleared was plotted vs. time, and the slope was estimated by linear regression. The slopes of clearance curves for the iBEC monolayer plus Transwell® membrane was denoted by PS_app_, where PS is the permeability × surface area product (in µL/min). The slope of the clearance curve for a Transwell® membrane without iBECs was denoted by PS_membrane_. The PS value for the iBEC monolayer (PS_e_) was calculated from 1/ PS_app_ = 1/ PS_membrane_ + 1/ PS_e_. The PS_e_ values were divided by the surface area of the Transwell® inserts (0.33 cm^2^) to generate the endothelial permeability coefficient (Pe, in µL/min/cm^2^), which was adjusted to units of nL/min/cm^2^.

### Bacterial heparinase I, II, and III treatments

Bacterial heparinase (HSase) I (Sigma, cat no. H2519), II (Sigma, cat no. H6512), and III (Sigma H6512) were obtained as lyophilized powders and stored at -20C in glass vials. These enzymes cleave the glycosidic bond between hexosamines and uronic acids of heparan sulfate, but each enzyme differs in its specificity largely based on sulfation patterns [26]. When used together, these enzymes can fully degrade heparan sulfate into its disaccharide components [27]. Stock solutions were prepared by reconstituting the enzymes in DMEM:F12 (Thermo Fisher Scientific, cat no. 11330032) + 0.1 mg/ml BSA and storing aliquots at −80°C. iBECs were treated in DMEM/F-12 medium (ThermoFisher, cat no. 11330032) + 1% B27 with HSase I, II, and III each at 0.25 U/mL (or vehicle) applied to the luminal side on day 8 for 24 h. The CCL2 transport assay occurred at t = 24 h on day 9.

### Tetra-acetylated N-azidoacetylglucosamine (GalNAz) treatments

Tetra-acetylated N-azidoacetylgalactosamine (GalNAz, Thermo Fisher Scientific, cat no. 88905), used to metabolically label glycosaminoglycans and glycoproteins, was obtained as a powder and stored at −20°C in a glass vial. Stock solutions were prepared by diluting GalNAz in DMSO according to kit instructions and were stored at −20°C. iBECs were then treated with a complete medium change of heparin-free DMEM:F12 + 1% B27 plus 40µM GalNAz (or DMSO vehicle) on day 3 and 100µM GalNAz or vehicle on day 6 post-subculture. We ascertained that the DMEM:F12 medium change did not affect TEER. The effects on CCL2 transport and HS content were assessed on day 9 post-subculture according to protocols described above.

### GalNAz click chemistry

To probe for GalNAz incorporation into iBEC HS chains, iBECs plated on 12-well plates were treated with GalNAz (or DMSO vehicle) on days 3 and 6 post-subculture in DMEM/F-12 + 1% B27 as described above. On day 8, GalNAz or DMSO-treated iBECs were further treated with HSase I, II, and III (all at 0.25 U/mL) for 24 h. On day 9, iBECs were washed in ice-cold PBS and scraped in PBS containing 1% TX-100 supplemented with protease (Sigma, cat no. P8340) and phosphatase (Sigma, cat no. P5726) inhibitors. Lysates were frozen at −80°C, then thawed on ice and centrifuged at 20000xg for 5 min at 4°C. The supernatant was saved, and protein concentrations were determined by Bradford assay (Thermo Fisher Scientific, cat no. 23200) using known concentrations of BSA to create the protein standard curve. For each well, 25 µg of protein was used for the click reaction with a biotinylated alkyne using the Click-iT™ Biotin Protein Analysis Detection Kit (Thermo Fisher cat no.C33372). Click chemistry was performed according to the manufacturer’s instructions. After the reaction, proteins were precipitated with chloroform and methanol, resolubilized in LDS sample buffer (Novex cat no. NP007) and denatured at 70°C for 10 min. Protein mixtures containing 5 µg of protein per well were electrophoresed using ExpressPlus PAGE precast gels (GeneScript, cat no. M41210). The proteins were then electrotransferred to a nitrocellulose membrane (Invitrogen, cat no. IB301002) using the iBlot Dry Blotting System (Invitrogen). Nonspecific binding was blocked by incubating the membranes in Tris-buffered saline 0.1% Tween-20 (TBS-T) supplemented with 5% BSA (Sigma, cat no. A7030) at RT for 45 min. The membrane was incubated for 2 h at 4°C in 5% BSA/TBS-T containing a 1:20000 dilution of poly-HRP streptavidin (ThermoFisher, cat no. N200). The membrane was washed with TBS-T three times for 5 min each. West Pico chemiluminescence reagent (Thermo Fisher Scientific cat no. PI-34078) was applied, and GalNAz-biotin-streptavidin linkages were visualized with the Amersham Imagequant 800 (Cytiva Life Sciences).

### iBEC RNA isolation and cDNA synthesis

Two independent differentiations of iBECs grown in subculture for 8–9 days in 12-well plates were lysed in 200ul/well Qiazol and RNA was isolated from a 4-well pool using RNeasy Lipid Tissue Mini Kit (Qiagen cat no. 74804) according to manufacturer’s instruction. 200ul of chloroform was added and the tube was shaken vigorously for 15 s. Tubes were incubated for 5 min at room temperature and centrifuged at 12,000g for 15 min at 4°C. The upper aqueous layer was transferred to a new tube and 1 vol of 70% ethanol was added and transferred to RNeasy Mini spin column and centrifuged. Spin column was washed with 700ul of buffer RW1 and 2 more washes of buffer RPE. 25ul of water was added on spin column, incubated at room temperature for 5 min and centrifuged to collect RNA. Nanodrop was used to assess RNA quality and quantity. The cDNA was made from iBECs using iScript™ Reverse Transcription Supermix for RT-qPCR (Bio Rad cat no. 1708840) according to manufacturer’s instruction. Briefly 1 ug of RNA was mixed with iScript RT supermix and incubated in a thermal cycler by priming at 25°C for 5 minutes, reverse transcription for 20 min at 46°C and RT inactivation at 95°C for 1 minute.

### Human microvessels cDNA synthesis

Human brain microvessels were isolated from the superior parietal lobule, which was dissected and immediately placed in 4°C endothelial cell (EC) media (Science Cell Research Laboratories, Catalog #1001) with 5% FBS (Science Cell Research Laboratories, Cat. #0025) for subsequent microvessel isolation as described previously [28]. All tissues were derived from brains donated for research from participants in the University of Washington (UW) BioRepository and Integrated Neuropathology (BRaIN) laboratory and precision neuropathology core, which supports a number of studies including the Pacific Northwest Brain Donor Network and research brain donors for the BRAIN Initiative Cell Atlas Network (BICAN), the UW Alzheimer’s disease research center (ADRC), the Kaiser Permanente Washington Health Research Institute (KPWHRI) Adult Changes in Thought (ACT), and others. All brain donations are performed with informed consent obtained during life under protocols approved by the Institutional Review Board (IRB) at UW and KPWHRI and/or after death with the legal next of kin. For this specific study, the UW Human Subjects Division deems the use of pre-existing de-identified samples as non-human subjects research. Fresh human brain samples were collected at the time of brain procurement and processing in donors with postmortem interval (PMI) <18h and cryopreserved [20,21]. All microvessels were assessed via light microscopy under 100x magnification to confirm the purity of each microvessel isolation. With each de-identified sample, relevant demographic and study data including age, sex, race, APOE genotype, dementia diagnosis, and summary neuropathologic data were provided for this study (see Table S1 in S1 File for complete details). Cryo stored MVs were thawed and incubated for 3 days before cDNA synthesis. Power SYBR® Green Cells-to-Ct™ Kit (Invitrogen cat no. A35379) was used to produce DNase 1 digested cell lysates and perform cDNA synthesis according to manufacturer’s instruction. Cells were lysed in 50ul of lysis solution with DNase 1 and incubated at room temperature for 5 minutes. 5ul of stop solution was added and incubated at room temperature for 2 minutes. cDNA was made by adding 25 ul of 2X SYBR RT buffer and 2.5 ul 20X RT Enzyme mix and 27.5 ul of lysed cell lysates and incubating in thermal cycler at 37°c for 60 minutes (reverse transcription hold), 95°C for 5 min (RT inactivation).

### RT-PCR

RT-PCR was performed using Viia7 Real-Time PCR system with SYBR Green Master Mix (Invitrogen cat no. A35379) for mRNAs corresponding to human CCR2, Syndecan 1–4, glypican 1–6, and two housekeeping genes, Ribosomal Protein L13, and Actin. The primers used for RT-PCR are shown in Table S2 in S1 File. Each sample (two independent iBEC differentiations and 3 human donors) was run in quadruplicate, and all genes were amplified on the same 384 well plate. Fluorescent signals were analyzed during each of 40 cycles consisting of denaturation (95°C, 15 s) and annealing (60°C, 1 minute).

### Statistics

The Prism 9.3 statistical software package was used for all statistical calculations (GraphPad Inc, San Diego, CA). For all figures, means are displayed with their standard deviations (SD). Linear regression lines and their slopes and intercepts were calculated using the Prism 9.3 software. Unpaired two-tailed t-tests were used to compare two means and analysis of variance (ANOVA) followed by Tukey’s multiple comparisons test when more than two means were compared.

## Results

### HS levels in iBECs are increased over time spent in culture

**Fig 1:**
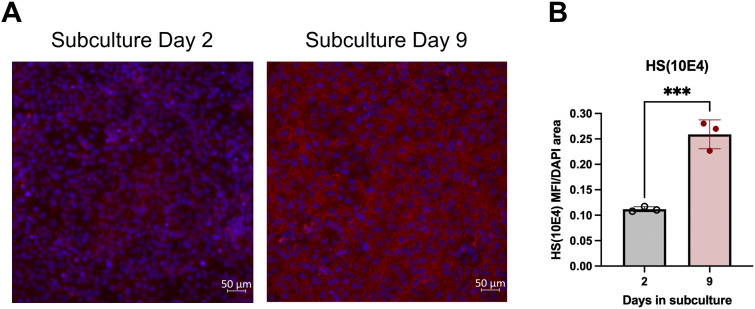
Heparan sulfate deposition in iBECs over time in subculture. a. Representative immunocytochemistry of Heparan Sulfate clone F58-10E4 (HS(10E4)) (red) on days 2 and 9 after subculture, counterstained with DAPI (blue). b. Immunofluorescence analysis of HS(10E4) mean fluorescence intensities (MFI) relative to DAPI area on days 2 and 9 after subculture. Each data point represents the average of 3 random fields of view per well. One differentiation was performed with n = 3 wells imaged. ***p < 0.001 (Unpaired two-tailed t-test). Means are displayed with their SD. Figure created with BioRender.

To establish whether iBECs are a suitable model to investigate the role of brain endothelial glycocalyx HSPGs in the blood-to-brain transport of CCL2, we first characterized their HS levels via immunocytochemistry using the anti-Heparan Sulfate clone F58-10E4 (HS(10E4)) antibody, which detects N-sulfated glucosamines of HS. We chose to quantify HS at an early (two days) and late (9 days) time point post-subculture, to determine whether HS accumulates over time in culture, as has been demonstrated with other in vitro endothelial models [19]. We have found previously that BBB integrity as measured by TEER and leakage markers does not change between these two time points of study, although other functional aspects such as glucose transport and cellular proliferation are downregulated after 9 days post-subculture [23]. Immunofluorescent analyses of HS(10E4) staining of iBECs at these time points identified a small amount of HS in iBECs at day 2 post-subculture which was significantly increased at day 9 post-subculture (Fig 1a–b), demonstrating that HS deposition occurs in iBECs with prolonged culture. We thus chose 9 days post-subculture as a timepoint in studies below to evaluate CCL2 transport across iBECs.

### Quiescent iBECs express a saturable blood-to-brain CCL2 transport system

**Fig 2:**
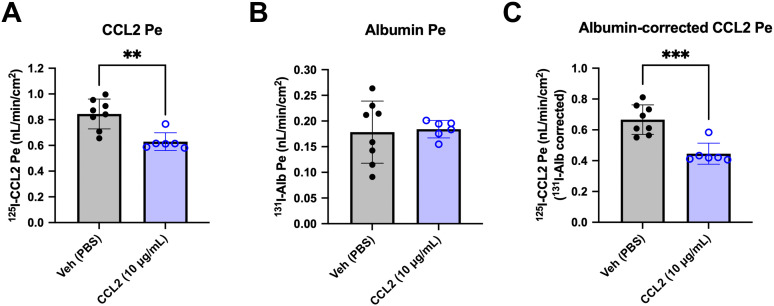
Saturability of CCL2 transport in iBECs. a. ^125^I-CCL2 Pe in the presence of 10 µg/mL CCL2 compared to PBS vehicle. b. ^131^I-Alb Pe in the presence of 10 µg/mL CCL2 compared to PBS vehicle. c. ^125^I-CCL2 Pe corrected for non-specific leakage as quantified by ^131^I-Alb Pe in the presence 10 µg/mL CCL2 compared to PBS vehicle. a-c. One differentiation was performed with n = 7-8 transwells per group. **p < 0.01, ***p < 0.001 (Unpaired two-tailed t-test). Means are displayed with their SD.

In contrast to BBB cytokine transport systems, saturability of chemokine transport has not been demonstrated *in vivo* [8,9,29–31]. However, HS binding sites on the glycocalyx of the endothelial cell lumen are presumed to be abundant, and so may require more unlabeled chemokine than can be practically used *in vivo* to saturate the system. We thus determined whether a very high/extraphysiological concentration of unlabeled CCL2 could inhibit transport of ^125^I-hCCL2. 10 µg/mL CCL2 significantly lowered ^125^I-CCL2 Pe (Fig 2a) but did not cause BBB disruption as evidenced by the lack of significant effect on ^131^I-Alb Pe (Fig 2b). The mean ^131^I-Alb-corrected ^125^I-CCL2 Pe was also significantly lowered relative to vehicle (67% of vehicle; 0.446 ± 0.028 nL/min/cm^2^ versus 0.666 ± 0.034 nL/min/cm^2^, respectively) (Fig 2c). These data support that saturable transport of ^125^I-hCCL2 can be measured *in vitro*.

### Detection of CCR2 and glycocalyx HSPG mRNA in iBECs and human brain microvessels

**Fig 3:**
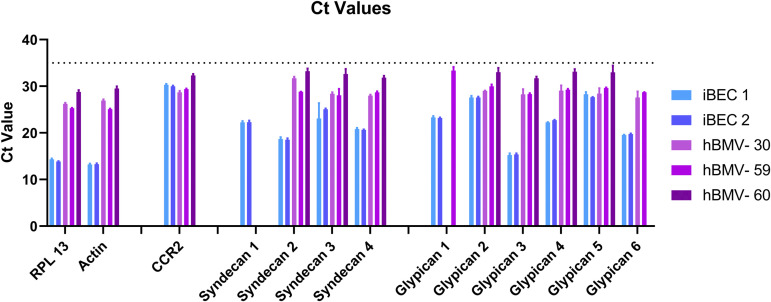
Ct values of qPCR data detecting housekeeping genes (RPL13, Actin), CCR2, and the glycocalyx HSPGs syndecan 1-4, and glypican 1-6. Data are showing the means of 3-4 technical replicates ± SD. The dotted line represents the Ct value cutoff of 35. Missing bars for a given transcript indicate the absence of reliable detection by qPCR. The numbers following “iBEC” in the legend indicate the experimental replicate. The numbers following “hBMV” indicate the age of the donor. As the main goal of this study was to show the presence or absence of a given transcript in each tissue, no statistical analysis was performed. Means are displayed with their SD.

To confirm that CCR2 and glycocalyx HSPGs are expressed in iBECs after 9 days in subculture, we isolated mRNA and performed qPCR in two independent differentiations of iBECs, alongside human brain microvessels (hBMVs) isolated from three different adult donors (age range 30–60) without a clinical diagnosis of dementia or brain pathology consistent with Alzheimer’s disease. All three donors had some degree of atherosclerosis or arteriolosclerosis. These and other demographics are shown in Table S1 in S1 File. The Ct values for housekeeping genes (RPL13 and β-actin) were higher for iBECs vs. hBMVs, likely reflecting biological differences in the relative proportion of proliferating vs. quiescent cells that is higher in iBECs [23,32–34]. Due to these differences in housekeeping genes and because our main goal was to determine the presence or absence of gene expression, we report and interpret raw Ct values rather than those normalized to housekeeping genes. Samples with Ct values ≥35 in more than one technical replicate were classified as “not detected,” and their bars were omitted from Fig 3. Results in Fig 3 show that iBECs express CCR2 at Ct values within the range of hBMVs, supporting that CCR2 is expressed, albeit at low levels, in both tissue types. Additionally, both iBEC differentiations expressed detectable levels of all known glycocalyx HSPGs, including syndecans 1–4 and glypicans 1–6. In comparison, syndecan-1 was not detected in any of the hBMV samples, which is consistent with prior bulk RNAseq analysis of human and mouse brain microvessels [35]. Glypican 1 and 6 were only detected in 1 or 2 hBMV samples, respectively, whereas glypicans 2–5 were detected in all hBMV samples. Prior bulk RNAseq analysis of glypicans in human and mouse BMVs was more variable, with glypican 1 and 5 consistently detected at low levels and the remainder being detected at very low levels in some subjects, but not others [35].

### Blood-to-brain transport of CCL2 in iBECs is inhibited by heparin, but not competitive inhibition of CCR2

**Fig 4:**
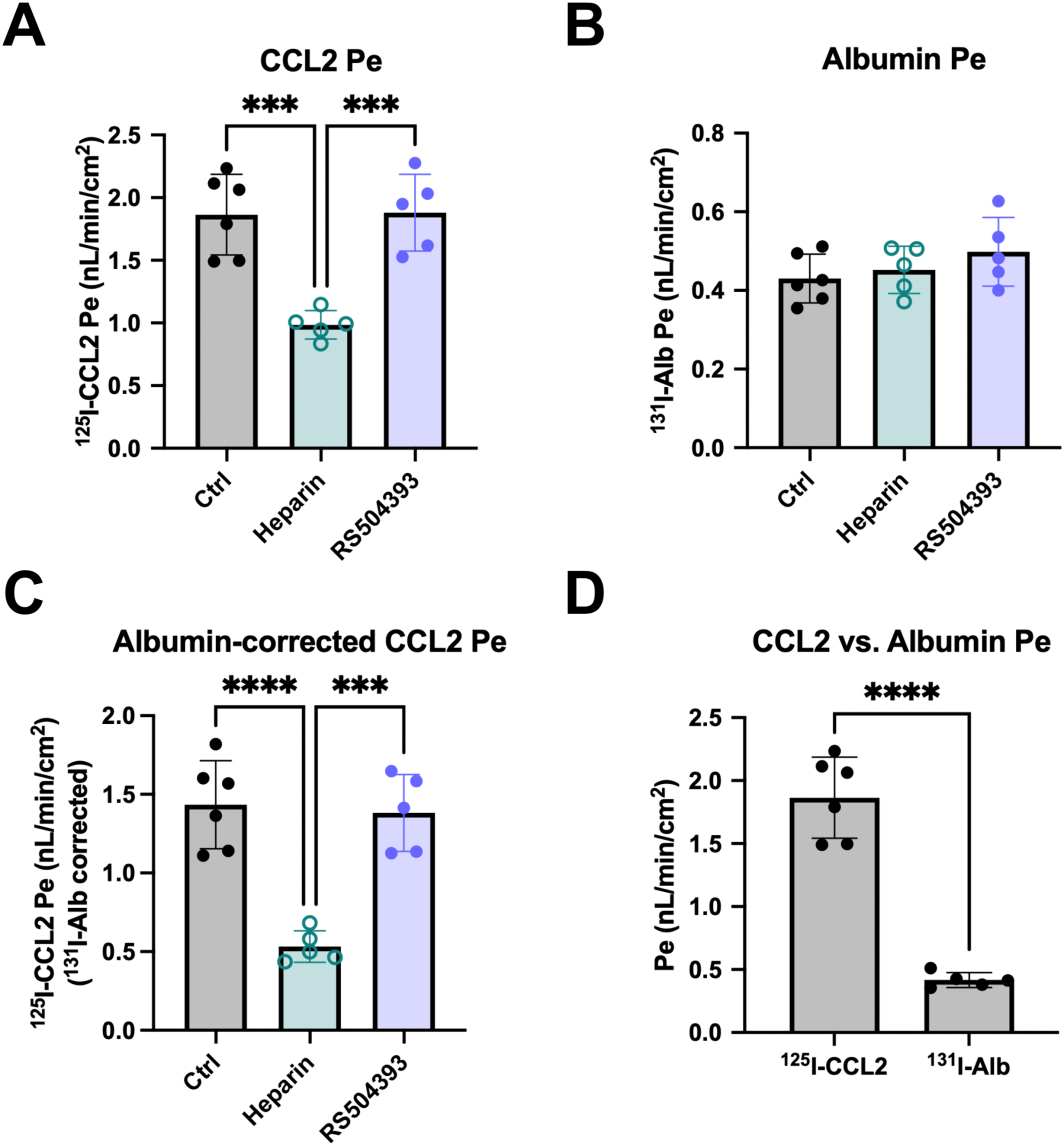
Effects of heparin and CCR2 antagonist on CCL2 transport in iBECs. a. ^125^I-CCL2 Pe in the presence of 20 U/mL heparin or 10 µM RS504393 (CCR2 antagonist) compared to control (“Ctrl”). b. ^131^I-Alb Pe in the presence of 20 U/mL heparin or 10 µM RS504393 compared to an untreated control group. c. ^125^I-CCL2 Pe corrected for non-specific leakage as quantified by ^131^I-Alb Pe in the presence of 20 U/mL heparin or 10 µM RS504393 compared to an untreated control group. a-c. ***p < 0.001, ****p < 0.0001 (One-way ANOVA with Tukey’s multiple comparisons test). d. Comparison of ^125^I-CCL2 and ^131^I-Alb Pe in the untreated control group. ****p < 0.0001 (Unpaired two-tailed t-test). a-d. One differentiation was performed with n = 5-6 transwells per group. Means are displayed with their SD.

We compared the transport of ^125^I-CCL2 Pe (Fig 4a) and ^131^I-Alb Pe (Fig 4b) at baseline (“Ctrl”) and in the presence of either 20 U/mL of heparin, a highly sulfated HS variant used to competitively inhibit interactions with HSPGs [36,37], or 10 µM of the CCR2 antagonist RS504393, which inhibits binding of human CCL2 to CCR2 [38] (Fig 4a–b). The concentrations of both inhibitors are equivalent to those used to evaluate inhibition of CCL2 transport across the mouse BBB *in situ* [39]. For each transwell, we subtracted the ^131^I-Alb Pe from the ^125^I-CCL2 Pe to exclude permeability due to leakage (Fig 4c). The addition of heparin produced a significant decrease in mean ^131^I-Alb-corrected ^125^I-CCL2 Pe relative to baseline (37% of Ctrl; 0.533 ± 0.045 nL/min/cm^2^ versus 1.43 ± 0.114 nL/min/cm^2^, respectively). The mean in the presence of RS504393 (1.38 ± 0.109 nL/min/cm^2^) at a concentration over 100-fold higher than the IC50 for CCR2 inhibition was approximately the same as the mean of the control group. At baseline, the mean ^125^I-CCL2 Pe was significantly greater than that of ^131^I-Alb (Fig 4d), supporting that CCL2 passage across iBECs is not due to non-specific leakage. These data support that iBECs possess a functional CCL2 transport system that is dependent on interactions with HS, but not interactions with CCR2, in agreement with our prior studies in mice [9].

### Transport of ^125^I-CCL2 is inhibited by heparinase enzymes applied to the luminal side

**Fig 5:**
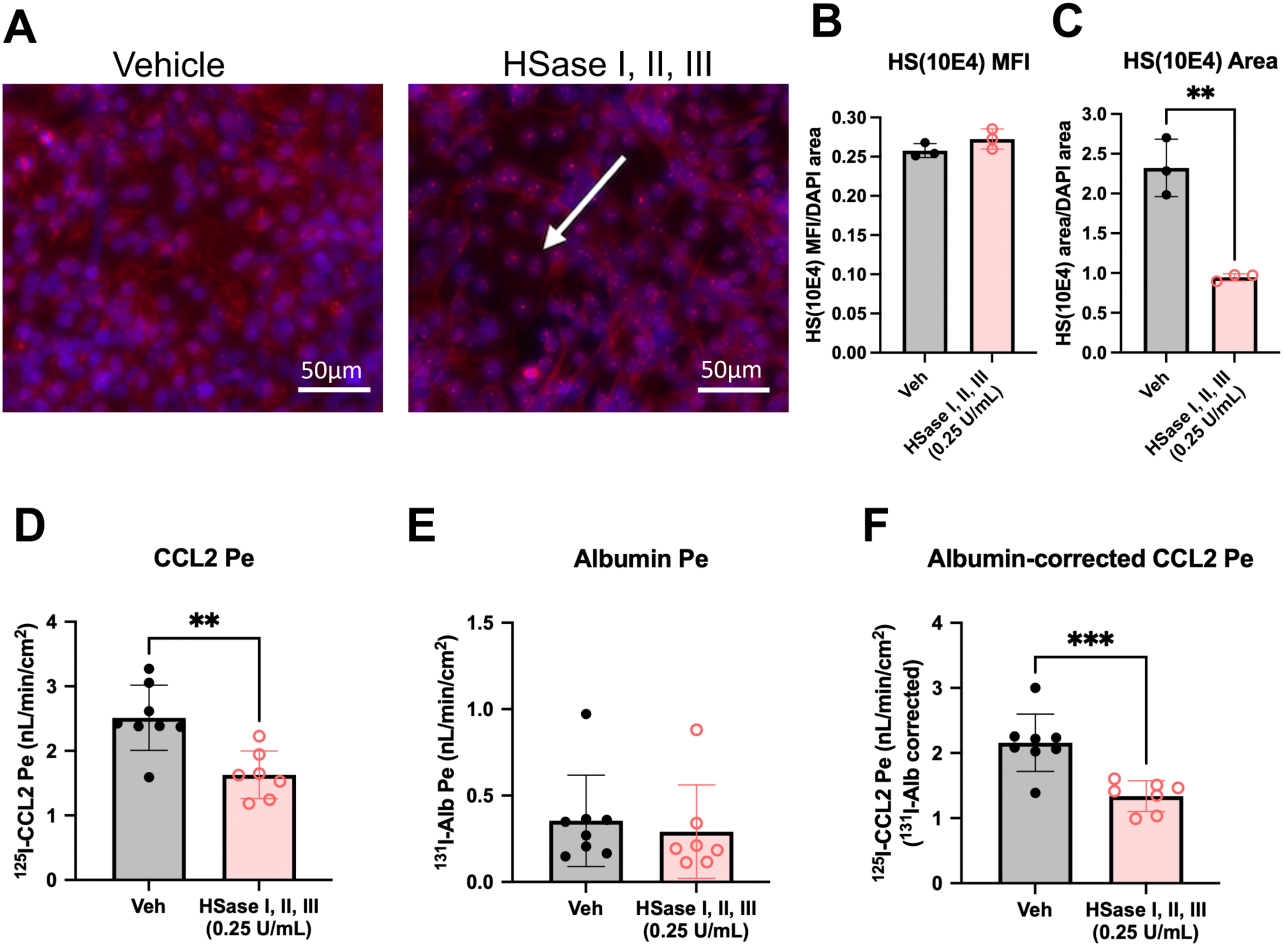
Effects of HSase treatment on CCL2 transport. a. Representative immunocytochemistry of Heparan Sulfate clone F58-10E4 (HS(10E4)) (red) and DAPI (blue) in vehicle or HSase I, II, and III-treated iBECs. b-c. Immunofluorescence analysis of HS(10E4) mean fluorescence intensities (MFI) relative to DAPI area (b) or total HS(10E4) area relative to DAPI area (c). d-e. ^125^I-CCL2 (d) or ^131^I-Alb in the presence of 0.25 U/ml HSase I, II, and III compared to vehicle. f. ^125^I-CCL2 Pe corrected for non-specific leakage as quantified by ^131^I-Alb Pe in the presence of 0.25 U/ml HSase I, II, and III compared to vehicle. One differentiation was performed with n = 7-8 transwells per group. *p < 0.05, ***p < 0.001 (Unpaired two-tailed t-test). Means are displayed with their SD. Figure created with BioRender.

To further evaluate the involvement of HS on CCL2 transport across iBECs, we determined whether treatment with bacterial heparinases (HSases) altered CCL2 transport kinetics. iBECs were treated on the luminal side only with HSase I, II, and III for 24 h. This combination of HSases in cell-free systems has been shown to completely degrade heparin and HS into its disaccharide components [40]. We first showed that our treatment with HS enzymes reduced the amount of HS that was detected by the 10E4 antibody (Fig 5A). The mean fluorescence intensity of HS normalized to DAPI area did not significantly change (Fig 5B), which was apparently due to residual intracellular/nuclear HS that remained post-digestion [41]. However, when we compared the HS area stained normalized to DAPI area, there was a significant decrease in signal following HSase enzyme treatment (Fig 5C), consistent with the degradation of extracellular HS. Enzymatic degradation of HS also inhibited CCL2 transport (Fig 5D–F), where the ^131^I-Alb-corrected ^125^I-CCL2 Pe was 62% of the vehicle control upon HSase treatment (2.16 ± 0.441 nL/min/cm^2^ versus 1.338 ± 0.236 nL/min/cm^2^, respectively). Importantly, ^131^I-Alb Pe was unaffected by HSase treatment, indicating that HSase treatment did not cause iBEC leakage. We also found that HSase treatment did not significantly affect TEER (Veh = 4483 ± 337.1 vs. HSase = 5071 ± 198.7, p = 0.15550), supporting that tight junctions remained intact with treatment.

### GalNAz treatment inhibits CCL2 transport in iBECs without decreasing total HS

**Fig 6:**
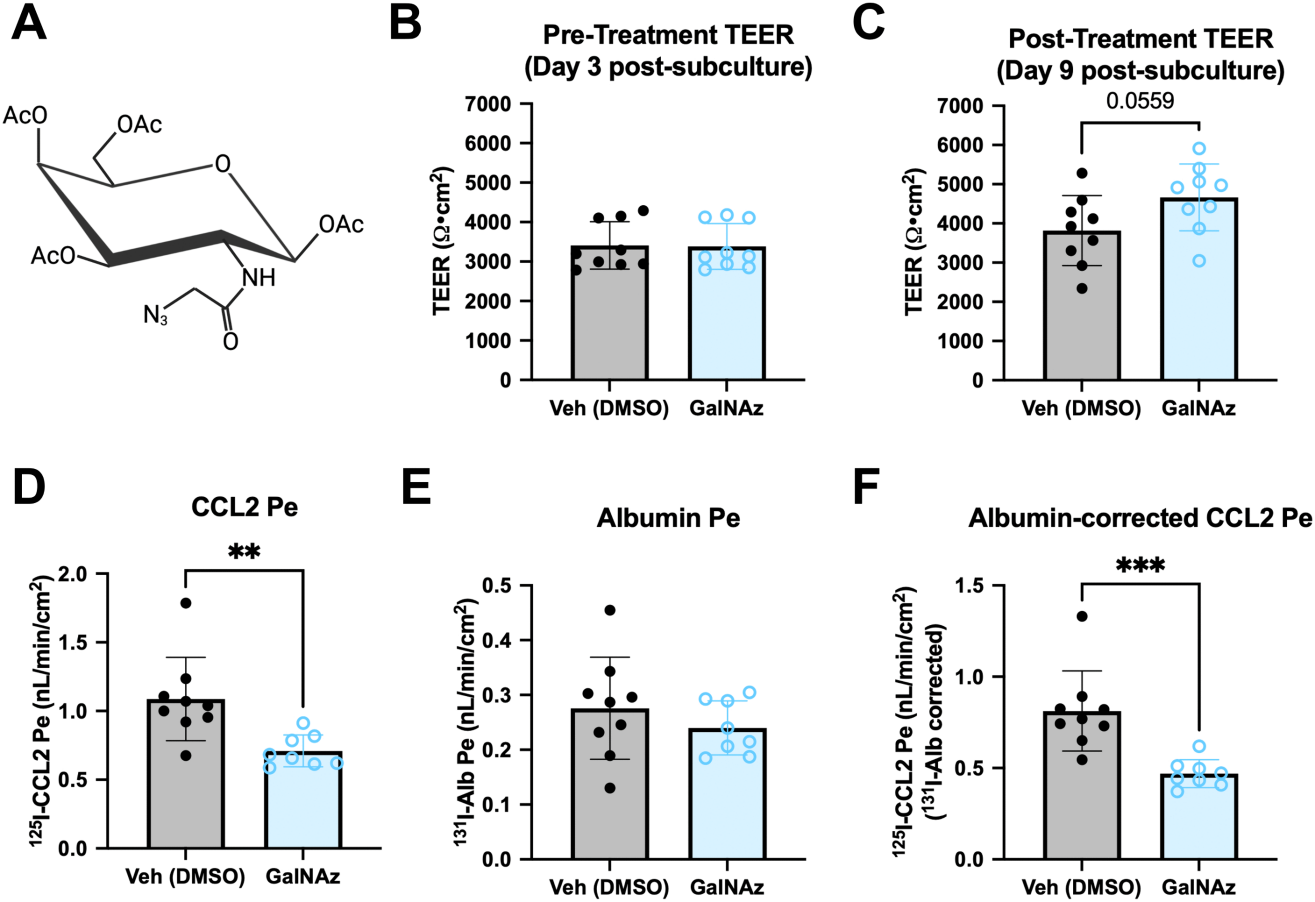
Effects of GalNAz on TEER and CCL2 transport in iBECs. a. Molecular structure of GalNAz. b. On day 3 post-subculture, transwells were organized into treatment groups such that TEER means were approximately equal and treated with GalNAz or DMSO (vehicle control).Transwells were re-treated on day 6 post-subculture, and the effects on TEER (c), ^125^I-CCL2 Pe (d) and ^131^I-Alb Pe (e) were measured on day 9 post-subculture. f. For each transwell, ^125^I-CCL2 Pe was corrected for non-specific leakage as quantified by ^131^I-Alb Pe. b-f. **p < 0.01, ***p < 0.001 (Unpaired two-tailed t-test). One differentiation was performed with n = 9 transwells treated per group. Means are displayed with their SD. Figure created with BioRender.

**Fig 7:**
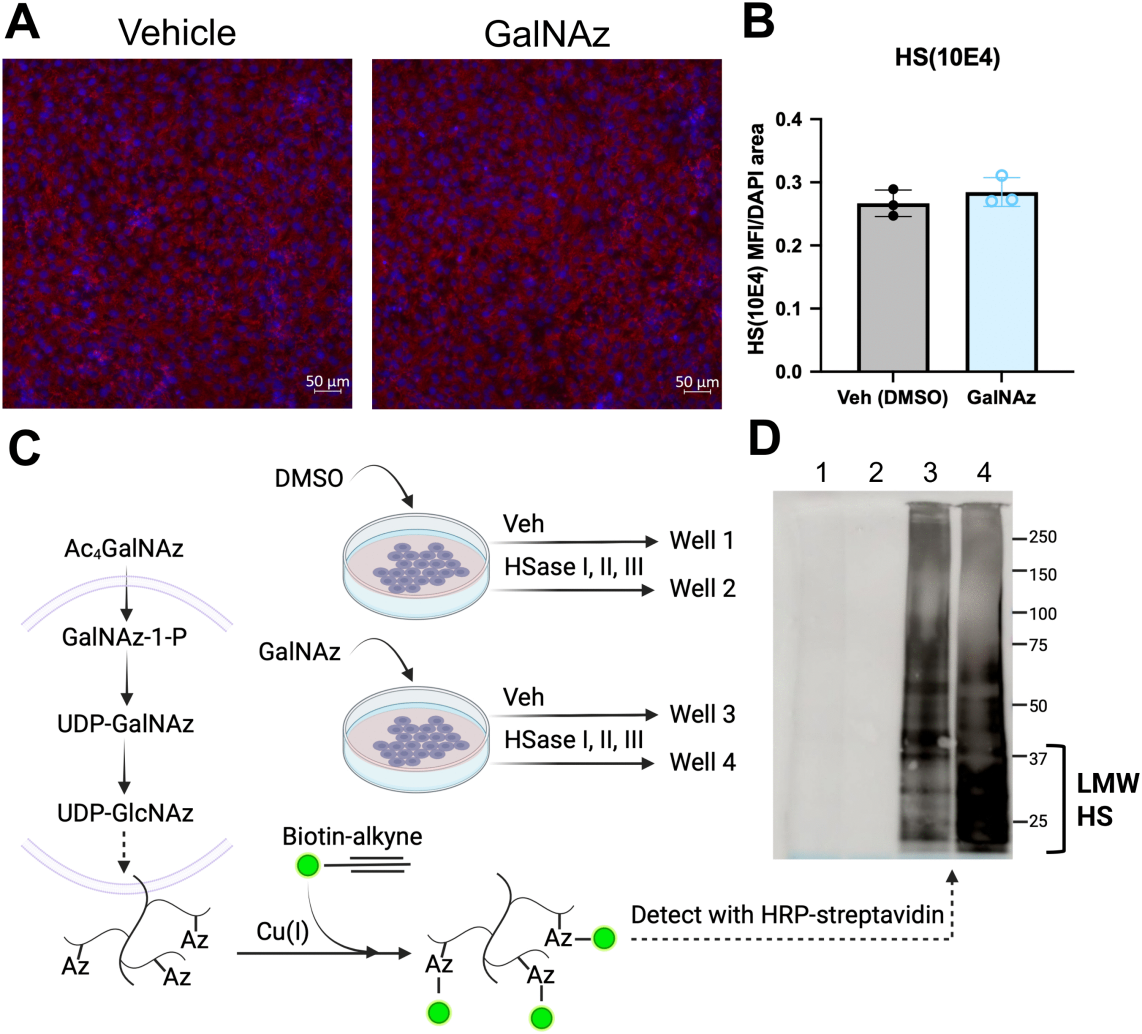
Effects of GalNAz treatment on HS(10E4) signal in iBECs. a. Immunofluorescence analysis of HS(10E4) mean fluorescence intensities (MFI) relative to DAPI area in iBECs on day 9 post-subculture after GalNaz treatment. Each data point represents the average of 3 random fields of view per well. One differentiation was performed with n = 3 wells imaged per differentiation. Means are displayed with their SD. b. Representative immunocytochemistry of HS10E4 (red) mean fluorescence intensities (MFI) relative to DAPI (blue) area. c. Graphic depicting the incorporation of azido groups into HS chains via the GalNAz salvage pathway, biotin labeling with click chemistry, and experimental design of GalNaz and HSase treatments for determination of GalNaz incorporation into HS chains. iBECs were treated with GalNAz or DMSO vehicle on days 3 and 6 post-subculture. On day 8, iBECs were further treated with either HSase I, II, and III (all at 0.25 U/mL) or vehicle. iBEC lysates were collected on day 9. d. Streptavidin blot detecting biotin-labeled HS incorporation into iBEC HS chains. Lanes 1 and 2 are blank, indicating specificity of biotin label for GalNaz-treated cells. Lane 3 depicts GalNaz labeling without HSase treatment, and Lane 4 depicts GalNaz labeling with HSase treatment, which resulted in an appearance of low-molecular weight (LMW) HS. Figure created with BioRender.

Tetra-acetylated N-azidoacetylgalactosamine (GalNAz), whose structure is shown in Fig 6a, was recently identified as a selective inhibitor of HS biosynthesis in CHO cells and zebrafish [42]. We thus tested whether GalNaz could inhibit CCL2 transport across the BBB using a subchronic treatment paradigm beginning at day 3 post-subculture, which we reasoned would optimally inhibit HS accumulation. We then measured TEER, ^125^I-CCL2 Pe and ^131^I-Alb Pe on day 9 post-subculture. We found that GalNAz treatment caused a small, but non-significant increase in TEER (Fig 6b–c) and did not alter ^131^I-Alb Pe (Fig 6e), indicating that it did not cause BBB leakage. GalNAz significantly reduced CCL2 Pe (Fig 6d) and decreased ^131^I-Alb-corrected ^125^I-CCL2 Pe from 0.812 ± 0.073 nL/min/cm^2^ to 0.470 ± 0.027 nL/min/cm^2^ (58% of vehicle). We next aimed to confirm that GalNAz reduced CCL2 transport by reducing HS levels, but surprisingly found that it did not decrease HS (10E4) antibody signal (Fig 7a–b), suggesting that GalNAz does not inhibit CCL2 transport by blocking HS synthesis in iBECs.

### GalNAz metabolically incorporates into iBEC HS chains

We reasoned that if GalNAz does not block HS synthesis in iBECs, it may reduce CCL2 transport by incorporating into HS chains, potentially causing structural modifications that inhibit CCL2 transport. GalNaz is a mimetic of N-acetylgalactosamine (GalNac), a precursor for chondroitin sulfate. However, the biosynthetic enzymes in the GalNAc salvage pathway, galactokinase 2 and GlcNAc-1-P uridyltransferase, can catalyze its phosphorylation and uridylation of GalNAz to UDP-GalNAz. UDP-galactose-4-epimerase can then epimerize UDP-GalNAz to UDP-GlcNAz, which mimics the UDP-N-acetylglucosamine (GlcNAc) required for HS biosynthesis [43]. Through “click” chemistry [44], which entails the chemoselective ligation of an azide and an alkyne labeled with biotin, azide-modified sugars can be detected (Fig 7c). To test GalNaz incorporation into HS in iBECs, we treated iBECs with GalNAz, and then treated with HSase I, II, and III (all at 0.25 U/mL) or vehicle on day 8 and collected iBEC lysates on day 9 at t = 24 h. iBEC lysates were then treated with a biotin-labelled alkyne that labels azido groups incorporated into glycosaminoglycan chains, separated on SDS-PAGE, and immunoblotted with HRP-linked streptavidin (Fig 7c). We found that cells treated with GalNAz were metabolically labeled, as evidenced by positive immunoreactivity for biotin (Fig 7D). Biotin signal in Lane 3 (Fig 7D) appears as a smear due to the varying lengths of HS chains and their attachment to proteoglycans of different sizes. HSase treatment resulted in the appearance of low-molecular weight biotin-labeled species (Fig 7D, lane 4), which are likely to be incompletely digested HS fragments generated by the enzyme cleavage. These findings support that GalNAz gets modified to GlcNAz and is subsequently incorporated into HS chains in iBECs. As the azido group on GlcNAz alters the chemical composition and likely the structure of HS, we posit that the mechanism of CCL2 transport inhibition by GalNaz occurs via HS structural modification rather than inhibition of HS synthesis.

## Discussion

Chemokines and their receptors constitute an elaborate signaling system that is responsible for orchestrating immune cell infiltration into the brain parenchyma and coordinating immune signaling within the CNS [10,45]. As chemokines are key mediators of neuroinflammation, it is important to determine the mechanisms that facilitate their accumulation and localized activities in the brain. In addition to local production by brain-resident cell types, blood-to-brain transport of circulating chemokines can occur and possibly contribute to aspects of the neuroinflammatory response, particularly relaying chemokines as signals of systemic inflammation to the brain [9] or recruiting cells like microglia to the brain vasculature [46]. Our recent work showed that CCL2 and CCL5 from the circulation can cross the intact mouse blood-BBB into the brain via a mechanism that involves their binding to heparan sulfate (HS), but not to chemokine receptors [9]. Results from the present study support that CCL2 transcytosis across iBECs occurs and is regulated by HS, in alignment with *in vivo* findings in mice.

HS is a predominant component of the BBB extracellular matrix, which comprises the basement membrane of the brain-facing side and the glycocalyx at the blood-facing side of brain endothelial cells [17,47,48]. Basement membrane HSPGs are mainly secreted, and include perlecan and agrin [48]. Because the HSPGs in the basement membrane are secreted and not membrane-attached, they are unlikely to function as transporters. Furthermore, it has been shown that CCL2 binding to the basement membrane/abluminal side of hBMVs is heparin sulfate-independent [18]. In contrast, glycocalyx HSPGs are mainly attached to the cell surface and include syndecans, which are transmembrane proteins, and glypicans, which are GPI-anchored proteins [17]. Both syndecans and glypicans have been shown to transport chemokines across peripheral vascular beds [49], and so are likely to be involved in the transport of CCL2. The function of syndecans and glypicans as transcytotic transporters of the BBB is underexplored. However, a few RNAseq studies have reported expression of syndecans 2–4 in brain endothelial cells [35,50–52], which we corroborate here in human brain microvessels via qPCR. Both human and mouse RNAseq datasets did not detect syndecan-1 [35], which is thought to predominate in the peripheral vasculature. The detection of relatively high levels of syndecan-1 in iBECs thus highlights a difference in HSPG expression vs. adult brain microvessels. Glypicans 1–6 are variably detected and generally expressed at lower levels than syndecans in the human brain microvasculature in RNAseq studies [32,35,52], and our qPCR data of hBMVs corroborate these findings. Our findings in iBECs show a more robust detection of glypicans 1–6 vs. hBMVs. Considering recent evidence that iBECs adopt some epithelial cell-like characteristics [53,54] and evaluated HSPG expression in compiled RNAseq datasets of primary endothelial and epithelial cells, as well as iBECs [54]. We found that iBECs differentiated using similar protocols had detectable expression of all syndecan and glypican HSPGs, and that levels were generally higher when compared with primary endothelial cells. However, primary epithelial cells had variable expression of HSPGs depending on the tissue type. For example, choroid plexus epithelial cells invariably expressed syndecan-2, and glypicans 3, 4, and 6, whereas colon epithelial cells invariably expressed all HSPGs except glypican-5. This indicates that the moderate expression of syndecan-1 is not a universal feature of epithelial cells. In contrast, we found that that syndecans 1–4 and glypicans 1–6 are expressed at moderate levels in undifferentiated iPSCs [55]. Therefore, we speculate that the differences in HSPGs for hBMVs vs iBECs may be due to residual gene expression in iBECs from the undifferentiated iPSC line. Future work is needed to evaluate the contribution of particular glycocalyx HSPGs to BBB transport of substances. We also recognize that modeling the HS composition of the BBB glycocalyx may be improved as a future direction through the development of iBECs that are closer phenotypically to brain endothelial cells, but that also retain high TEER that is necessary to study transport processes.

In addition to HSPGs, we examined the expression of CCR2 in iBECs and hBMVs to assess whether it was detectable by qPCR. CCR2 mRNA was detected in both iBECs and hBMVs at high Ct values, consistent with low transcript abundance in both tissue types. Although RNAseq datasets of hBMVs and iBECs often do not detect CCR2, this method is less sensitive for detecting low-abundance transcripts, which explains why CCR2 is detected in some RNAseq datasets [32], but not others [52,54]. Further, even though brain endothelial CCR2 mRNA is expressed at very low baseline levels, evidence supports that CCR2 has important functions in brain endothelial cells [56]. Ultimately, our results support that CCR2 does not contribute to CCL2 transport in iBECs, as demonstrated by the lack of effect from a pharmacological inhibitor, and possibly also due to low levels of CCR2 expression.

Our findings that HS accumulates in iBECs over time in culture highlights the utility of using this particular BBB model for prolonged culture studies [21]. It has been shown that the extracellular matrix, and particularly the glycocalyx of endothelial cells, accumulates with prolonged culture [19], consistent with our results. A limitation of this study is that we did not evaluate CCL2 transport in iBECs over time in culture as the glycocalyx develops. Results of such studies may be difficult to interpret because iBECs undergo many phenotypic changes over time in culture that include reduced proliferation, maturation of tight junctions, and altered expression and function of transporters like GLUT1 [23]. Therefore, differences in CCL2 transport observed over time could be due to something other than HS (e.g., post-uptake transport machinery). The more compelling evidence for HS involvement comes from the three complementary approaches we employed: competition with heparin, heparinase digestion, and GalNaz treatment. However, future work investigating CCL2 transport in iBECs over time could provide further insight into the regulation of HS ligand transport as the glycocalyx develops. Regarding disease relevance, recent work has shown that HS deposition is reduced in iBECs derived from patients with genetic risk for schizophrenia [57], supporting that iBECs could be used in future studies to investigate aspects of extracellular matrix dysregulation in neurological diseases. Notably, we used iBECs in a static culture format. We selected this model due to the relative ease of designing rigorous, moderate-throughput transport studies in which compartments are readily accessible for radioligand measurement. However, it is supported that glycocalyx structure, particularly its thickness and mechanotransduction properties, more closely recapitulate those of the vasculature in vivo when flow is introduced to the system [17,19,58]. Even so, HS-dependent CCL2 transport occurred in the iBEC static culture model, and this may be due to involvement of cell surface-bound HS which is in close proximity to the luminal membrane. In contrast, the more distal components of the brain endothelial glycocalyx, such as hyaluronan, have been shown to contribute to diffusion barrier properties to larger molecules [59]. Basement membrane HSPGs such as agrin have also been shown to contribute to BBB maintenance and integrity; in the case of agrin this is via stabilization of adherens junctions, which lowers the paracellular leakage of endothelial cells [60]. However, in our studies, neither GalNaz nor HSases applied to the luminal side of iBECs compromised barrier properties. Future studies are needed to determine how flow-dependent maturation of the iBEC glycocalyx impacts BBB integrity and transport of CCL2 and other substances that use HS to cross the BBB.

To directly probe the role of HS in CCL2 transport, we treated iBECs with bacterial heparinases (HSases) and assessed both HS abundance and CCL2 transport activity. With our fixed-cell, permeabilized staining method, anti-HS antibody access to both surface-exposed and intracellular HS epitopes is unlikely to be confounded by factors such as antibody internalization, rerouting, or degradation that can occur in live-cell staining. Because the accessibility of HS cleavage sites is likely limited to some extent by the complex structure of HS, complete HS degradation is not expected using this approach. We observed that mean fluorescence intensity of HS remained unchanged whereas the total area of HS coverage was reduced, consistent with focal rather than uniform degradation of HS. Such a pattern could arise if some epitopes were fully degraded while others persisted due to steric hindrance or other barriers to enzyme activity, leaving a residual HS pool that may still contribute to CCL2 binding or transport. Consistent with this, CCL2 transport across iBECs was significantly reduced, but not completely ablated by heparinase treatment. We did not evaluate whether HSase treatment results in the upregulation of CCL2. Whereas this could be an applicable issue in more complex model systems, it is unlikely that increased CCL2 production by iBECs would have resulted in measurable competitive inhibition of its transport in our experiments. This is because our methods entail replacing the culture medium immediately prior to initiating transport assays, and so no endogenous CCL2 or HSase is present when transport assays are initiated. Additionally, the short timeframe of our transport assays would only allow minimal CCL2 accumulation, and very high concentrations of recombinant CCL2 (10 ug/mL) were needed to observe saturable transport, suggesting that HS has a very high capacity for transport.

A notable limitation of our study design was that each individual transport experiment was conducted using a single independent differentiation of iBECs from the GM25256 hiPSC line. However, the dataset as a whole encompasses seven independent differentiations, with no figure containing data from the same differentiation as another figure. This design ensures that our central findings are not dependent on a single differentiation outcome. All experiments in this study were conducted using a rigorously characterized differentiation method with high fidelity [23]. Some expected variation was observed in the absolute Pe values across differentiations, albeit within an acceptable range (e.g., the albumin Pe coefficient of variation across experiments is 35% and albumin-corrected CCL2 Pe coefficient of variation is a bit higher at 53%). Importantly, results across independent differentiations support the robustness of the conclusion that CCL2 transport is HS-mediated in iBECs from GM25256 iPSCs. Future work is needed to determine whether iBECs from other iPSC lines are equally suited for studies of HS-mediated transport.

HS and other sulfated glycosaminoglycans contribute to the overall negative surface charge of the BBB luminal surface, and are likely to contribute to aspects of protein binding and transport. Adsorptive transcytosis is one mechanism of BBB transcytosis that occurs when positively charged molecules bind the negatively charged luminal surface and are subsequently internalized via energy-dependent, vesicular processes [61]. The recombinant human CCL2 used here has an isoelectric point of 9.39 (as determined by Expasy), and so is positively charged at physiological pH. However, interactions of CCL2 with HS and heparin are well characterized and depend on select basic amino acid residues within specific sites of the protein sequence rather than the net positive charge [62]. It’s supported that although positive charge contributes to the ability of substances to cross the intact BBB, many aspects such as endocytic routing post-internalization are nuanced. For example, cell penetrating peptide conjugates are variably routed to lysosomes for degradation depending on the attached cargo [61]. Importantly, we controlled for degradation here by only measuring CCL2 and albumin that crossed the BBB intact. Given the apparent complexity, future work is needed to elucidate the mechanisms of HS-dependent transcytosis across the BBB.

## Conclusions

To date, just a few studies have evaluated the contributions of HS in the transport of substances other than chemokines across the BBB [36,37,63–66]. These include findings of HS involvement in the brain uptake of HIV and its proteins [36,65,67] and of exosomes [37], brain clearance of amyloid beta [64], and the uptake of basic-fibroblast growth factor by the basement membrane HSPG perlecan [66]. Together with our present findings, these studies support an important but underexplored role of BBB HS in the regulation of BBB transport and iBECs as a human *in vitro* model that allows for future studies of HSPG transporter functions.

## Supporting information

S1 File Supplementary Tables 1 and 2. Excel files containing human brain microvessel donor information (Table S1) and primer sequences for qPCR studies of Fig 3 (Table S2).
(XLSX)
